# Decision-making regarding social situations in people with intellectual disability at different stages of the decision-making process

**DOI:** 10.1007/s10339-024-01193-1

**Published:** 2024-05-07

**Authors:** Agnieszka Fusinska-Korpik, Michal Gacek

**Affiliations:** 1 Faculty of Medicine and Health Sciences, Andrzej Frycz Modrzewski Krakow University, ul. Herlinga-Grudzinskiego 1, 30-705 Krakow, Poland; 2 Institute of Psychology, Jagiellonian University, ul. Ingardena 6, 30-060 Krakow, Poland

**Keywords:** Intellectual disability, Social decision-making, Decision-making process, Assessment

## Abstract

Decision-making capability is essential in fulfilling the need for autonomy of people with intellectual disability. In this study we aimed to examine decision-making capability regarding important social situations in people with intellectual disability at different stages of decision-making process. We studied 80 vocational school students with mild intellectual disability and 80 students of a similar age from mass vocation schools. We assessed decision-making with Important Life Decisions Task (ILDT). Students with intellectual disability obtained significantly lower scores than controls for each of the stories in ILDT as in each stage and overall final score in the decision-making process. The magnitude of difference in scores between groups varied in different stages of decision-making process. The most notable difficulties in decision-making regarding important social situations in people with intellectual disability are related to the evaluation of alternatives stage. Pattern of differences obtained in our study may be related to the content of decision-making problems.

## Background

Current social policies towards people with intellectual disability manifest recognition of the fact that people with this type of disability should have the right to exert control over their lives setting their own goals and making their own decisions and choices (Curryer et al 2015; United Nations 2006). Opportunities to make decisions and choices regarding one’s life are essential in fulfilling the need for autonomy, and as such are an important aspect of self-determination (Burke et al. 2020; Deci and Ryan 2000; Wehmeyer and Abery 2013). The results of studies involving people with intellectual disability indicate that autonomy predicts positive outcomes in employment and independent living in this group and that it is also positively related to quality of life (Lachapelle et al. 2005; Neely-Barnes et al. 2008; Shogren and Shaw, 2016). However, a major concern regarding the autonomy of people with intellectual disability is associated with shortcomings in the decision-making process which increase the risk of acting in a way that may be detrimental or even harmful to the person (Hickson and Khemka 2014). Decisions made in an impulsive or superficial manner may lead to dire consequences for people with intellectual disabilities, possibly resulting in victimization, abuse or sexual exploitation (Khemka et al. 2009).

The complexity of the decision-making process is reflected in the Pathways of Decision Making model, proposed by Hickson and Khemka (2013; 2014). This model builds on the results of studies on people with intellectual disability; as such, it can be particularly useful as a framework for assessment of decision-making in this group (Hickson and Khemka 1999; Khemka and Hickson 2006). According to this model, decision-making is influenced by cognitive, motivational and emotional mental processes that may be related to different situational or environmental contextual factors and to the biological and developmental characteristics of the decision-maker. The Pathways of Decision Making model recognizes different ways of making decisions that may be related to intuitive processing, which is rapid and automatic, and to reasoned processing, which requires a more calculated and cognitively demanding approach from a person. When describing the reasoned type of decision-processing, Hickson and Khemka take inspiration from the developmental approach towards decision-making (Byrnes 1998), incorporating the idea that rational decision-making should be viewed as a four-step process (Byrnes et al. 1999; Byrnes 2002). First, a person needs to identify the problem or set a goal. In the second step, the person needs to generate options that could potentially be helpful in achieving the goal or solving the problem. In the third step, the person needs to evaluate the generated alternatives. Finally, in the fourth step, the person needs to select one of these alternatives. The four-stages approach has proved useful in revealing developmental differences in participants from the general population (Byrnes et al. 1999).

Studies on decision-making in people with intellectual disability cover a wide array of types of decision-making situations, focusing on such topics as financial decisions (Suto et al. 2005; Willner et al. 2010), situations involving risk of abuse (Khemka and Hickson 2000), maternal care (Tymchuk et al. 1990), cyberbullying (Khemka and Hickson 2017), coercive situations (Khemka et al. 2009), medical decisions (Wong et al. 2000), employment related decisions (Timmons et al. 2011), and social and interpersonal decisions varying in level of complexity (Burke et al. 2019; Smith 1986; Jenkinson and Nelms 1994). Typically, in these studies decision-making is assessed using vignettes on which decision-making situations are described and asking a person to make a decision (e.g. Jenkinson and Nelms 1994; Khemka et al. 2009; Suto et al. 2005). In some studies authors use other methods, such as computer tasks, like those used in a study on temporal discounting (Willner et al 2010), or interviews subjected to qualitative analyses (Timmons et al. 2011). Although due to differences in design of decision-making studies generalisations regarding functioning of people with intellectual disability need to be made with caution, some results seem to be consistent across the reports. One of the most consistent results of previous studies concerns the fact that comparison of people with intellectual disability to controls from the general population indicates that decision-making capability is less effective in the former group, which is usually explained in relation to differences in cognitive functioning (e.g., Hickson et al. 1998; Jenkinson and Nelms 1994). However, it is important to note that decision-making is not determined solely by cognitive abilities, but it is also related to emotional and motivational factors which may interact with cognitive processes (Hickson and Khemka 1999; 2013); also, some studies have found that individual scores of people with intellectual disability are quite varied, and some participants are able to make decisions which are as good as those of controls (e.g., Suto et al. 2005; Tymchuk et al. 1990). It is often noted that results in decision-making tasks indicate difficulties in predicting and evaluating long-term consequences of actions in people with intellectual disability, and a tendency to respond impulsively which in certain types of situations may lead to heightened risk of abuse and victimisation (Jenkinson and Nelms 1994; Khemka and Hickson 2017; Willner et al. 2010).

Studies on social decision-making in people with intellectual disabilities reveal that participants in this group exhibit fewer types of problem-solving strategies and use less strategies that reflect higher levels of social understanding than participants matched for chronological age (Smith 1986). Also, it has been shown that participants with intellectual disability propose a limited number of solutions which are often irrelevant to the problem at hand (Wehmeyr and Kelchner, 1994) and give more maladaptive responses, which suggests a tendency towards impulsive and hasty actions or overreactions, especially when it comes to important social decisions (Jenkinson and Nelms 1994). Studies which required making decisions regarding potentially harmful social situations have shown that people with intellectual disabilities have difficulties in suggesting prevention-focused actions in response to simulated situations of abuse (Khemka and Hickson 2000; Khemka et al. 2009). Typically, studies on social decision-making in people with intellectual disabilities do not focus on the whole decision-making process but only on the ‘generation of alternatives’ stage of decision-making (for a comprehensive review, see Hickson and Khemka 1999 or Hickson and Khemka 2014).

The aim of the present study was to examine decision-making capability regarding social situations in people with intellectual disability who attended special vocational schools in Poland. In our study we examined decision-making in people with intellectual disability at different stages of the decision-making process, in comparison to a group of controls from the general population. Also, a new decision-making measure developed for the purpose of our research is described here in order to make it available for other researchers and practitioners. Our study was based on a study of financial decision-making in people with intellectual disability where patterns of performance at different stages of decision-making process were presented (Suto et al. 2005). Research in which the stages of the decision-making process are examined expand the understanding of the decision-making difficulties experienced by people with intellectual disability. However, the content of decision-making tasks may be important for decision-making performance (Blais and Weber 2001; Pachur and Spaar 2015; Weller et al. 2015), and the results of studies on financial decisions may not apply to other types of decisions. Thus, we decided to examine the stages of the decision-making process in situations which may be potentially related to real-life dilemmas experienced by people with intellectual disabilities, such as decisions regarding choice of romantic partner, interactions with family members in difficult situations, or choice of vocational and employment path. These types of decisions are important for the autonomy and well-being of people with intellectual disability (Jenkinson and Nelms 1994; Timmons et al. 2011). Also, the performance of people with intellectual disabilities in such decision-making situations has not been investigated in regard to particular stages of the decision-making process.

## Method

### Ethical considerations

The study was approved by the ethical committee at the Pedagogical University of Krakow (approval number WP.113-1/19) and by the relevant school boards. Written informed consent was obtained from all participants and their parents.

### Participants

Results of power analysis indicated that two equal size groups with 64 participants are required to achieve a power of *β* = 0.80 and a mean effect of *d* = 0.5. In the study we assessed 80 young adults with mild intellectual disability who attended special vocational schools in Poland (*M*_*age*_ = 18.44; *SD*_*age*_ = 1.16; *Min*_*age*_ = 17; *Max*_*age*_ = 23; *M*_*IQ*_ = 65.56; *SD*_*IQ*_ = 5.42), and 80 controls from mass vocational education schools with no diagnosis of developmental disorders and a vocational profile similar to that of participants with intellectual disability (*M*_*age*_ = 18.26; *SD*_*age*_ = 0.9; *Min*_*age*_ = 16; *Max*_*age*_ = 23; *M*_*IQ*_ = 89.44; *SD*_*IQ*_ = 10.34). In the group of participants with intellectual disability, we assessed 40 boys (*M*_*age*_ = 18.55; *SD*_*age*_ = 1.08; *Min*_*age*_ = 17; *Max*_*age*_ = 21; *M*_*IQ*_ = 65.7; *SD*_*IQ*_ = 5.43) and 40 girls (*M*_*age*_ = 18.32; *SD*_*age*_ = 1.23; *Min*_*age*_ = 17; *Max*_*age*_ = 23; *M*_*IQ*_ = 65.42; *SD*_*IQ*_ = 5.49). In the group of participants from mass vocational schools, we assessed 41 boys (*M*_*age*_ = 18.34; *SD*_*age*_ = 1.02; *Min*_*age*_ = 16; *Max*_*age*_ = 23; *M*_*IQ*_ = 87.93; *SD*_*IQ*_ = 11.85) and 39 girls (*M*_*age*_ = 18.18; *SD*_*age*_ = 0.76; *Min*_*age*_ = 17; *Max*_*age*_ = 20; *M*_*IQ*_ = 91.03; *SD*_*IQ*_ = 8.32).

Intellectual disability is defined by significant limitations in intellectual functioning and adaptive behaviour which originate in the developmental period (American Psychiatric Association 2013; World Health Organization 2019); these essential criteria have been consistently used in different diagnostic classifications for the last 50 years (Tasse et al. 2016). In our study, we did not include people with conditions that often co-occur with intellectual disability, namely genetic disorders, combined disabilities, or psychopathology (Einfeld et al. 2011; Harris 2006; Mazza et al. 2020). In our study we aimed to form a relatively homogenous group, considering the fact that people with known genetic disorders, such as Down syndrome, possess behavioural phenotypes that may be related to specific social behavior (Dykens 2000; Goscicki et al. 2021); also, the co-occurrence of psychiatric conditions with intellectual disability may have a significant impact on social decision-making in this group (Mastilo et al. 2023). Each student with intellectual disability had a diagnosis provided by a psychological-pedagogical counselling centre based on the ICD-10 (World Health Organization 1996) criteria. No observations were excluded from the database before performing analyses. Information regarding participants’ IQ was obtained with the Wechsler Adult Intelligence Scale—Revised. Verbal and Full-Scale IQ scores in WAIS-R correlated significantly with the score on the Important Life Decisions Task used in the study, thus indicating the importance of cognitive processes for decision-making and supporting the validity of the measure used. The relationship between IQ scores and scores in the decision-making task used in our study has been described in detail in another article (Fusinska-Korpik and Gacek 2022).

### Measure

In this study we used a measure called *Important Life Decisions Task* (*ILDT*), which we developed to assess decision-making regarding social situations. Important life decisions, labelled as “major decisions”, were also assessed in a study by Jenkinson and Nelms (1994) but measurement with ILDT provides more detailed description of problem situations, and a more precise procedure on how to conduct and evaluate a semi-structured interview. We used a scoring system based on a study of financial decision-making by Suto et al. (2005). This scoring system allows separate scores to be obtained on a three-point scale for each of the four stages of the decision-making process, as well as a general decision-making score (see Appendix 2).

The measure used in our study comprised of six vignettes depicting social problem situations, and one extra training vignette, which was presented in the beginning of the task as an example. Descriptions of the situations in the vignettes were prepared using common vocabulary and simple sentences. Each vignette depicted a situation in which the protagonist has to make a choice and at least two options are presented, each option having its own advantages and disadvantages. The decisions concerned situations regarding which candidate to go out with on a date, which parent to spend time with in time of financial difficulties, how to act towards a partner of a divorced parent, what kind of vocational profile to choose at school, what kind of employment opportunity to choose, and what kind of place to choose to live in (see Appendix 1).

We assessed four stages of decision-making process: (1) identification of a problem, (2) generation of alternatives, (3) evaluation of alternatives, (4) and decision-making with a justification. Each time a researcher read a story and asked questions relevant to one of the stages of the decision-making process considered in this study: (1) what decision does the protagonist of the story face?; (2) what possible choices does he/she have?; (3) what are the advantages and disadvantages related to each choice?; (4) what decision should the protagonist make and why? After obtaining answers for questions (2) and (3) the researcher asked a follow-up question: “Is there anything else that comes to your mind? Are there any other alternatives/advantages or disadvantages?”. Follow-up questions were asked once per participant. In regard to question (4) if a participant gave the answer and did not provide a justification, the researcher asked: “Can you tell me why he/she should make this decision?”. Also, the researcher asked questions to clarify the answers, e.g. in story 1 “which boy/girl do you have in mind?”. If the participant gave information which was not relevant to the story, e.g. wrong names of the characters, or said that he or she cannot remember some facts, the researcher offered to read the story once again. Recurring mistakes were scored as inadequate answers. Each time the study began with an example vignette. If participants did not know how to answer the questions regarding this vignette, they were not tested further in the study. Exclusion for this reason occurred only for two participants with intellectual disability.

Each part of the decision-making process was evaluated on a scale from 1 to 3 points. 1 indicated the lack of relevant information, 2 indicated an incomplete answer, and 3 indicated a correct and comprehensive answer. Participants also obtained separate extra points in regard to the number of alternatives and number of advantages and disadvantages mentioned. We added one extra point for each correct choice mentioned by a participant and one extra point each time when a participant could explain why a choice might be beneficial or disadvantageous. In this article we present final scores on a 3-point scale, as well as scores for the sum of alternatives and the sum of advantages and disadvantages mentioned. Details regarding scoring criteria were presented in Appendix 2. The final score for each of the stories was calculated by adding points regarding the four aspects of decision-making process evaluated on a 3-point scale and the scores for all the alternatives and advantages and disadvantages mentioned by the participant. The total score in ILDT was calculated by adding the final score for each of the stories and dividing it by six.

### Procedure

All the students were recruited at schools after the study had been approved by the school boards. In special schools, school psychologists identified those students who did not fulfil the study’s exclusion criteria (i.e., diagnosis of genetic disorders, combined disabilities or psychopathology). The participants and their parents were later contacted by a psychology student regarding the study. The participants were tested individually at schools by one of the four female psychology students, who were given detailed instructions on the procedure and administration of the ILDT. All the participants were informed that they could withdraw from the study at any time without consequences. The answers in the ILDT were recorded and then transcribed by the principal investigator. For the answers they obtained during study, the psychology students who tested the participants proposed scores, which were then consulted with the head-researcher. In most cases the scoring was consistent. In rare cases of inconsistencies, the answers were discussed by the group of psychology students who conducted the study to justify the score.

### Statistical analyses

We performed analyses using R software, ver. 40.4 (R Core Team 2021). First, we calculated descriptive statistics for each of the stories in ILDT and for the total score, and for each of the four stages of the decision-making process in both studied groups. We obtained 95% bootstrap confidence intervals based on 10,000 resamples. We used the Shapiro–Wilk test to examine the distribution of the data. Next, we used the Wilcoxon rank-sum test with the Holm adjustment for multiple testing to compare results obtained by participants with intellectual disability and those from control group. We treated group as an independent variable, and the scores for each of the stories in ILDT and the total score, and the scores for each of the stages of the decision-making process as dependent variables. As a measure of the effect size we used rank-biserial correlation coefficients (Cureton 1956).

## Results

Participants with intellectual disability obtained lower mean scores than participants in the control group for each of the stories and for the total score, and for each of the decision-making stages. Since the scores were not normally distributed for each of the stories in both groups, except for Story 2 score in the group of people with intellectual disability, and for each of the decision-making stages, except for the generation of alternatives in the control group and sum of the scores for the alternatives in both groups, we decided to use nonparametric methods to compare the groups. The descriptive statistics for all variables considered in our study are presented in Table 1; the results of the Shapiro–Wilk test for both groups are presented in Table 2.

**Table 1:** Descriptive statistics for each of the stories, the total score in the Important Life Decisions Task, and for the aspects of the decision-making process assessed by this measure

		M(Median)	SD	Range	95%CI	Skew	Kurtosis
Story 1	Int.dis	16.27 (16)	4.18	[5, 27]	[15.53, 17.05]	0.34	3.41
	Controls	20.52 (20)	4,59	[10, 33]	[19.7, 21.39]	0.48	2.85
Story 2	Int.dis	15.71 (15.5)	5.02	[4, 34]	[14.78, 16.65]	0.36	4.48
	Controls	20.54 (20)	5.06	[12, 36]	[19.61, 21.45]	0.65	3.13
Story 3	Int.dis	12.95 (13)	5.51	[4, 30]	[11.94, 13.95]	0.56	3.34
	Controls	17.7 (18)	4.54	[4, 28]	[16.86, 18.51]	− 0.72	4.13
Story 4	Int.dis	16.2 (16)	2.93	[11, 24]	[15.66, 16.75]	0.3	2.76
	Controls	18.88 (18)	4.09	[12, 34]	[18.15, 19.64]	10.27	5.03
Story 5	Int.dis	14.97 (16)	4	[4, 24]	[14.24, 15.7]	− 0.32	3.45
	Controls	18.26 (18)	4.79	[5, 31]	[17.39, 19.14]	− 0.35	3.88
Story 6	Int.dis	16.9 (18)	4.1	[7, 29]	[16.15, 17.65]	− 0.07	3.4
	Controls	21.66 (22)	4.49	[13, 34]	[20.86, 22.49]	0.15	2.91
Total score	Int.dis	15.5 (15.5)	3.43	[8.8, 25.5]	[14.88, 16.14]	0.33	3.02
	Controls	19.59 (19.08)	3.66	[12.3, 29]	[18.92, 20.28]	0.26	2.65
Identification	Int.dis	15.21 (16)	2.55	[8, 18]	[14.72, 15.68]	− 10.01	3.43
	Controls	17.27 (18)	1.09	[13, 18]	[17.08, 17.46]	− 10.56	5.04
Alternatives	Int.dis	11.68 (12)	2.09	[8, 17]	[11.3, 12.06]	0.22	2.45
	Controls	13.36 (13)	2.08	[8, 18]	[12.97, 13.74]	0.05	2.65
Alternatives (sum)	Int.dis	13.44 (13)	2.73	[7, 20]	[12.95, 13.94]	0.23	2.82
	Controls	15.12 (15)	2.74	[9, 21]	[14.62, 15.61]	0.16	2.41
Evaluation	Int.dis	10.51 (10)	3.13	[6, 18]	[9.95, 11.09]	0.46	2.22
	Controls	14.55 (15)	2.78	[6, 18]	[14.03, 15.05]	− 0.79	3.57
Evaluation (sum)	Int.dis	25.8 (25)	11.52	[3, 73]	[23.76, 27.98]	10.11	5.56
	Controls	39.77 (36)	15.18	[13, 87]	[37.08, 42.59]	0.72	3.27
Decision	Int.dis	16.38 (17)	1.96	[11, 18]	[16.01, 16.73]	− 10.11	3.16
	Controls	17.48 (18)	1.14	[14, 18]	[17.25, 17.68]	− 10.68	5.34

*95%CI*—95% bootstrap confidence intervals

**Table 2:** Results of the Shapiro–Wilk test for each of the stories, the total score in the Important Life Decisions Task, and for the aspects of the decision-making process assessed by this measure

	Intellectual disability		Control group	
	W	*p*	W	*p*
Story 1	0.97	0.042*	0.96	0.007**
Story 2	0.97	0.069	0.96	0.015*
Story 3	0.97	0.032*	0.95	0.003**
Story 4	0.94	0.001**	0.9	< 0.001***
Story 5	0.97	0.029*	0.95	0.004**
Story 6	0.97	0.034*	0.97	0.035*
Total score	0.98	0.39	0.98	0.38
Identification	0.89	< 0.001***	0.7	< 0.001***
Alternatives	0.96	0.025*	0.97	0.055
Alternatives (sum)	0.97	0.1	0.97	0.053
Evaluation	0.94	0.002**	0.92	< 0.001***
Evaluation (sum)	0.94	< 0.001***	0.96	0.009**
Decision	0.8	< 0.001***	0.62	< 0.001***

*W*—the value of the Shapiro–Wilk statistic; *p*—probability value for the Shapiro–Wilk test; *p < 0.05, **p < 0.01, ***p < 0.001

**Fig. 1:**
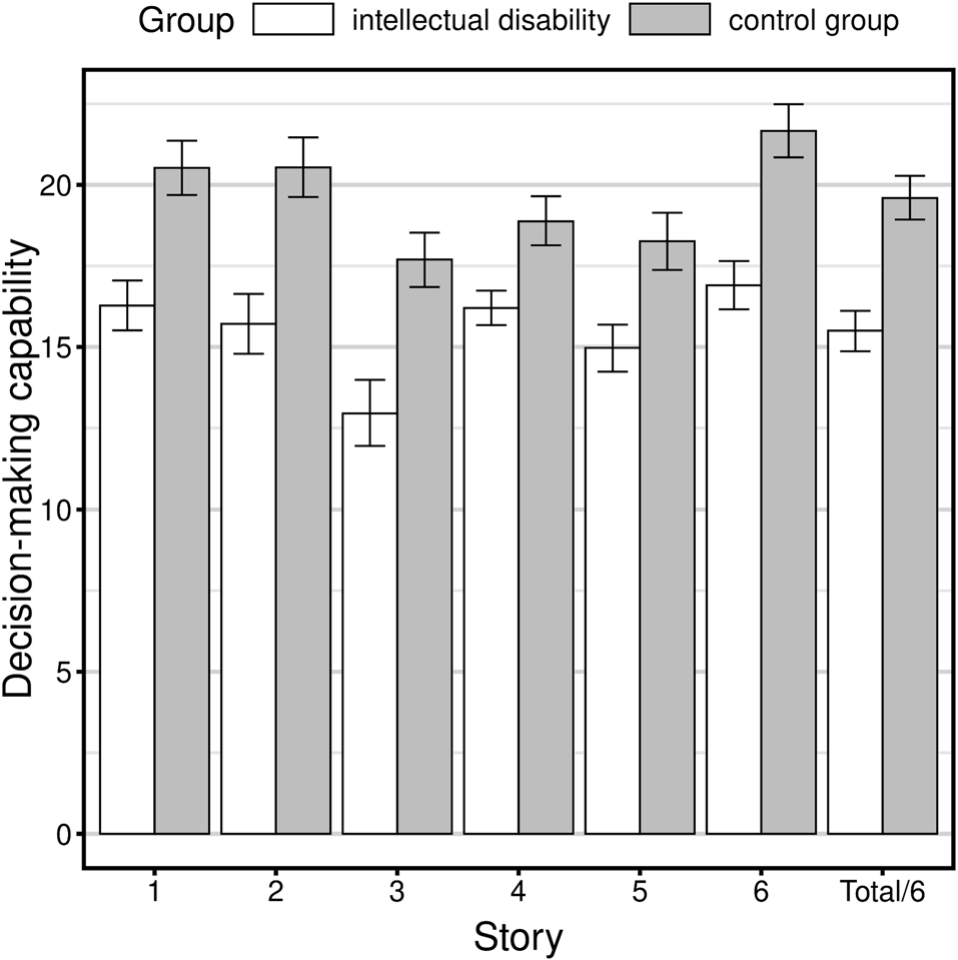
Graph showing the mean scores of participants with 95% confidence intervals on each story in Important Life Decisions Task and the total score

The mean scores in each of the ILDT stories ranged from 12.95 to 16.9 in participants with intellectual disability and from 17.7 to 21.66 in the control group. The lowest mean scores in both groups were obtained for Story 3 which depicted a situation in which the protagonist decides on how to act towards a new partner of a divorced parent. Comparison of results from both groups indicated that all the scores in the group of people with intellectual disability were significantly lower than those of controls (*p* < 0.001 for each of the stories and for the total score). Rank-biserial correlation coefficients indicate that the magnitude of difference in the mean scores was almost identical in regard to stories 1, 2, 3, and 6 (*r* ranging from 0.5 to 0.56), and slightly lower for story 4 (*r* = 0.37) and story 5 (*r* = 0.42). The results of the Wilcoxon test and rank-biserial coefficients are presented in Table 3. The results obtained in both groups for each of the stories and for the total score are graphically presented in Fig. 1.

**Table 3:** Results of the Wilcoxon test and rank-biserial coefficients for each of the stories, the total score in Important Life Decisions Task, and for the aspects of the decision-making process assessed by this measure

	W	*p*	r	95%CI
Story 1	1587.5	< 0.001***	0.5	[3, 5]
Story 2	1566.5	< 0.001***	0.51	[3, 6]
Story 3	1465.5	< 0.001***	0.54	[4, 7]
Story 4	2001.5	< 0.001***	0.37	[1, 3]
Story 5	1851.5	< 0.001***	0.42	[2, 5]
Story 6	1405	< 0.001***	0.56	[3, 6]
Total score	1322.5	< 0.001***	0.59	[3, 5.17]
Identification	1463	< 0.001***	0.54	[1, 2]
Alternatives	1831.5	< 0.001***	0.43	[1, 2]
Alternatives (sum)	2107	< 0.001***	0.34	[1, 3]
Evaluation	1125.5	< 0.001***	0.65	[3, 5]
Evaluation (sum)	1433.5	< 0.001***	0.55	[9, 17]
Decision	2073.5	< 0.001***	0.35	[0.000048, 1]

*W*—the value for the Wilcoxon test statistic; *p*—probability value for the Wilcoxon test corrected for multiple comparisons; *r*—rank-biserial correlation coefficients; *95%CI*—95% bootstrap confidence intervals for the effect based on the Wilcoxon test results, *p < 0.05, **p < 0.01, ***p < 0.001

**Fig. 2:**
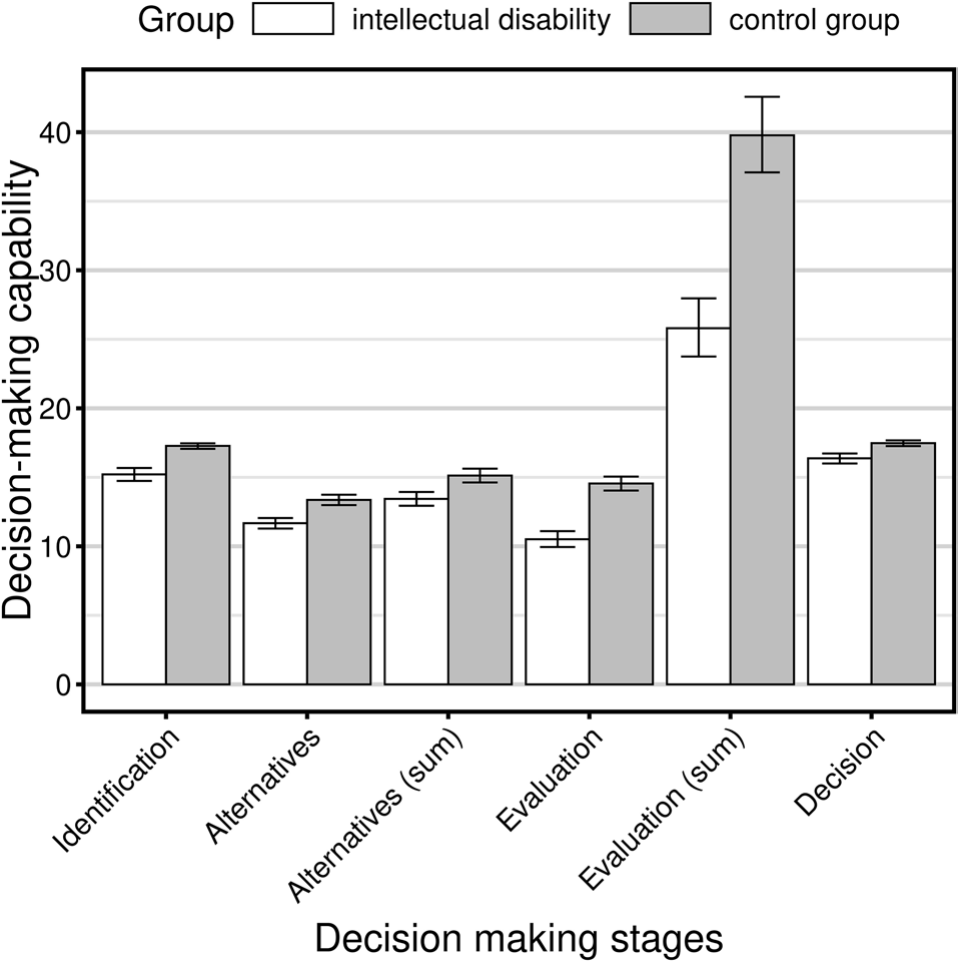
Graph showing the mean scores of participants with 95% confidence intervals obtained in Important Life Decisions Task in each of the stages of the decision-making process

Difference in scores in favour of the control group regarding decision-making ability was statistically significant for each of the four stages of decision-making process and for the number of generated alternatives, and the number of advantages and disadvantages mentioned (*p* < 0.001 for each result). Rank-biserial correlation coefficients ranged from 0.34 to 0.65 (see Table 3). Pattern of results indicates that difficulties in decision-making in people with intellectual disability vary in different stages of decision-making process. The highest correlation coefficient, indicating the most notable difference in performance, was related to evaluation of alternatives (*r* = 0.65 for this aspect, and *r* = 0.55 for the sum of alternatives). Next significant difference was related to identification of a problem (*r* = 0.54). Differences between people with intellectual disability and controls regarding generation of alternatives and making a justified decision were lower (*r* = 0.43 and *r* = 0.35, respectively). The scores regarding decision-making stages are graphically presented in Fig. 2. Quantitative disparities corresponded with certain qualitative differences in the given answers. Specifically, students from the control group usually mentioned advantages and disadvantages which related to more abstract and intangible concepts (e.g., “if he left home, he would be more independent”), while participants with intellectual disabilities gave answers that were related rather to specific losses or gains (e.g. “if he stays at home, his parents will cook for him”). Also, people with intellectual disabilities used less sophisticated vocabulary and were less precise in their answers than controls.

## Discussion

### Decision-making in people with intellectual disability

In our study we aimed to examine decision-making capability of people with intellectual disability at different stages of the decision-making process in comparison to a control group from the general population. We proposed a measure called Important Life Decisions Task comprising of vignettes on which decision-making dilemmas regarding social situations which may have serious consequences for the protagonist were described. The content of the stories was partially inspired by a measure used in a study by Jenkinson and Nelms (1994), and the scoring system was based on a study of financial decision-making by Suto et al. (2005). The results regarding ILDT indicate that participants with intellectual disabilities obtained similar mean scores in each of the stories, with the exception of the story about a divorced parent’s new partner, in which the obtained scores were the lowest in this group. The difficulties in obtaining higher scores in this story may stem from the fact that this problem situation was probably not related to an actual life situation or future expectancies of most participants, which could make it difficult to analyse it as thoroughly as those presented in other stories. Magnitude of difference in results between a group with intellectual disability and controls was similar in most stories, and slightly lower in stories regarding choice of vocational profile at school, and choice of employment opportunity. This may be associated with the fact that participants in both groups attended vocational schools, thus topics like vocational preparation and employment may be closely related to their experiences and discussions during classes.

The results obtained in our study are consistent with the findings of previous studies which indicated that people with intellectual disability experience difficulties in decision-making related to social situations (Hickson and Khemka 2014; Khemka and Hickson 2017). Significant differences in performance between people with intellectual disability and controls were revealed in each of the stages of the decision-making process. Such results are in accordance with results of other studies on social decision-making in which participants with intellectual disability proposed limited number of solutions to presented problems (Wehmeyer and Kelchner 1994) and gave more maladaptive responses (Jenkinson and Nelms 1994). However, the interesting result that we obtained was that the difficulties in decision-making seem to vary in different stages of a decision-making process. In our study the most notable difference between the studied groups occurred during the evaluation of alternatives, which indicates that pointing out advantages and disadvantages of alternatives is the most difficult part of the decision-making process for people with intellectual disability. The fact that evaluation of alternatives may be more difficult for people with intellectual disabilities than other stages of the decision-making process may relate to difficulties in abstract reasoning in this group. During the study we observed a tendency of people with intellectual disability to focus more on specific losses or gains related to practical aspects of functioning than on abstract concepts, such as personal independence. The second highest magnitude of difference in performance between the studied groups was obtained for the ability to correctly identify a problem in a decision-making situation. This result indicates that people with intellectual disabilities start to experience difficulties at the initial stage of the decision-making process, which might have a negative impact on performance in subsequent stages. Lesser magnitudes of differences were found for the generation of alternatives, and the ability to make a decision and justify it.

Pattern of difficulties experienced by participants with intellectual disability in our study differs from the pattern obtained in the study on financial decision-making by Suto et al. (2005). In this study people with intellectual disability had most difficulties during the part of the decision-making process labelled as “understanding”, which included both generation of alternatives and their evaluation. In our study, where separate scores for these two aspects of decision-making were used, we found that generation of alternatives seems to be less difficult for people with intellectual disability than evaluation of alternatives. This indicates that the two mentioned aspects of the decision-making process should be assessed as separate in future studies. The second aspect of decision-making that was most difficult for people with intellectual disability in the study by Suto et al. (2005) was labelled “reasoning”, and it was related to making a choice and justifying it. In this study the identification of a problem was less difficult for participants with intellectual disability than understanding and reasoning. Pattern of results in our study indicate that identification of a problem was the second most difficult stage of decision-making after generation of alternatives, and making a decision and justifying it was least difficult for our participants with intellectual disability. This suggests that decision-making content may elicit difficulties related to different stages of the decision-making process (Blais and Weber 2001; Pachur and Spaar 2015; Weller et al. 2015). It is possible that making a logical decision and justifying it may be easier for people with intellectual disability in the social than the financial context. This may be due to the fact that financial decisions involve more abstract and math-related issues, which may make it more difficult for people with intellectual disability to assess them properly.

### Limitations and implications for future research

Our study has several limitations related to the study design and the measure we used. Regarding the limitations related to our study design, it is important to note that we assessed only one type of decision-making situation and results in decision-making tasks may depend on the content of decision-making situations (Blais and Weber 2001; Pachur and Spaar 2015; Weller et al. 2015). In future it would be important to study decision-making processes by considering different types of situations in order to explore how the decision-making content relates to a decision-making performance in participants with intellectual disability. Second, in our study we did not include persons with known genetic syndromes, combined disabilities, and psychopathology. Such diagnoses are frequent in groups of people with intellectual disability (Einfeld et al. 2011; Harris 2006; Mazza et al. 2020), but also participants with such diagnoses may present specific patterns of performance in social decision tasks (Dykens 2000; Goscicki et al. 2021; Mastilo et al. 2023). In future, it is important to include such participants in similar studies. Also, we examined only students with mild intellectual disability who attended special vocational schools; the controls came from mass vocational schools. Assessing participants from other groups, e.g., those with above-average intelligence or with moderate intellectual disability, should give further information on the relationship between decision-making ability and intelligence.

The limitations regarding the measure we used concern the need to provide additional data regarding the reliability and validity of the ILDT. So far, we have confirmed that the results of the ILDT correlate with the Verbal and Full-Scale IQ scores in WAIS-R, which stands in accordance with the fact that rational decision-making requires the use of cognitive abilities which should be linked to intelligence (Fusinska-Korpik and Gacek 2022). However, there is a need to examine the ILDT in relation to measures that tap other aspects of functioning which may be related to decision-making, such as adaptive behaviour, or motivational and emotional processes. Also, personal experiences of people with intellectual disability related to different types of decision-making situations should be considered in future studies. Further studies with the use of the measure presented in detail in this article should make it possible to expand the knowledge regarding the decision-making process in people with intellectual disability. Also, the results obtained with the ILDT may be useful in developing programs to enhance decision-making abilities at different stages of the decision-making process. The results may be beneficial for practitioners who try to achieve a better understanding of students’ cognitive difficulties in order to consider them in their educational or therapeutical work.

### Conclusion

We examined the decision-making capability of people with intellectual disability at different stages of the decision-making process in comparison to a control group from the general population. People with intellectual disabilities presented more difficulties than controls in making decisions regarding important social situations at each stage of the decision-making process. The most notable difference in performance was observed in the ‘evaluation of alternatives’ stage of the decision-making process. The second highest magnitude of difference was observed in regard to identification of the problem in a decision-making situation. The obtained pattern of results differs from results regarding financial decision-making, thus suggesting that difficulties in the decision-making process should be studied and discussed in relation to the content of decision-making situations.

## Data Availability

The data supporting the findings of this study will be made available by the authors to qualified researchers upon reasonable request.

## Appendix 1

Vignettes included in Important Life Decisions Task.

## Example vignette

(Female protagonist for girls and male protagonist for boys).

Anne/Paul on Mondays starts her/his lessons at 8 o'clock in the morning. She/He set his alarm clock for 7 o'clock, but she/he overslept and there are only 15 min left before the start of the lesson. Anne/Paul is very sleepy and knows that she/he will be on time only for the second lesson. Anne/Paul’s parents are not home, so no one will know that she/he has stayed at home. But the girl/boy has several absences and is afraid of getting into trouble at school. Anne/Paul has to make a decision.

## Girl version

Claire met two boys, Tomek and Dominik, during a holiday trip. Claire is very attracted to Tomek because of his looks, but they don't have much in common to talk about. With Dominik, Claire could talk for hours, but his looks are less to her liking. Valentine's Day is coming. Both friends invite her to the cinema, and she plans to go with one of them. Claire has to make a decision.

## Boy version

Mike met two girls, Anne and Dominika, during a holiday trip. Mike is very attracted to Anne because of her looks, but they don't have much in common to talk about. With Dominika, Mike could talk for hours, but her looks are less to his liking. Valentine's Day is coming. Mike’s parents gave him some money and he wants to invite one of his girlfriends to the cinema. Mike has to make a decision.

### Story 2

Due to a difficult financial situation, Kate/Matthew's mother had to go abroad for work. Kate/Matthew hasn't seen her for a few months and her/his mum suggests that she/he joins her for holidays together. Kate/Matthew misses her/his mum. Also, mum lives right by the sea and Kate/Matthew could have a great holiday. Kate/Matthew's dad has not been feeling well lately and her/his help around the house is much needed. Dad reassures her/him that he is well and agrees for her/him to go, but Kate/Matthew is worried that he won't be able to manage on his own and will feel lonely. There is an aunt near Kate/Matthew's house who could visit Dad, but she will not have time to visit very often. Kate/Matthew has to make a decision.

### Story 3

Magda/Mark's parents divorced a year ago. The girl/boy lives with her/his mother and sees her/his father once a week. Despite the divorce she/he has a very good relationship with both parents. Her/his mother has been seeing a new man for some time. Magda/Mark does not like him because she/he would like her/his parents to be together again. Mum asked Magda/Mark if he/she had any objections to her new partner moving in with them permanently. Magda/Mark would prefer that Mum waited with such a big decision. She/he is afraid to talk to her about it because she/he does not want her to be angry with her/him. Magda/Mark knows that mum's new partner is good for her, and mum feels happy with him, but she/he finds it difficult to accept him. She/he knows that if she/he doesn't agree to live together, mum will respect that and the two of them will continue to live together alone. Magda/Mark has to make a decision.

### Story 4

Marta/Greg is finishing middle school this year and plans to start a vocational school. She/he has always liked cooking very much. That is why she/he is thinking about studying in a ‘cooking’ or ‘confectionery’ class. There are several restaurants and canteens in Marta/Greg's town, so it will be easier for Marta/Greg to find a job as a cook. However, she/he knows from her/his friend that the wages are very low. The earnings of a confectioner are a bit higher, but there is only one pastry shop in his/her town, so it will be difficult for her/him to find a job. Marta/Greg has to make a decision.

### Story 5

Joan/Philip is finishing vocational school this year. She/he has always dreamed of becoming a cook and this has influenced her/his choice of school. The mother of Joan/Philip's friend owns a bar and promises to hire her/him for an unpaid internship. Joan/Philip would gain experience and new skills. At the same time Joan/Philip gets an offer to take a paid job as a salesman in a neighbouring village. Joan/Philip would have to commute to work every day. So, she/he will have to get up very early and return home late in the evening. Joan/Philip's parents are not doing very well, so she/he would like to help them financially. Joan/Philip has to make a decision.

### Story 6

Caroline/David found a well-paid job six months ago. She/he currently lives in a small flat with her/his parents and younger sister. She/he has a very good commute from her/his family home. The girl/boy is thinking more and more about becoming fully independent and renting a flat just for herself/himself. Caroline/David has already looked at several flats. Two of them she/he particularly likes, even though they are far from her/his workplace. One flat is newly renovated and fully furnished. It also has a separate kitchen and is very comfortable. Unfortunately, the rent is quite high, and Caroline/David would have little money left to live on their own. The second flat is much smaller, consisting of one room connected to the kitchen. It is much cheaper but is not fully furnished. Caroline/David would therefore have to spend part of their savings on household equipment. The girl/boy could also stay in the family house and give up independent living. Caroline/David has to make a decision.

## Appendix 2

Framework for semi-structured interview, and scoring system, in the assessment of Important Life Decisions Task.

**Table:**

Decision-making stages	Scoring system	
	Score	Scoring criteria
Identification (what decision does the protagonist of the story face?)	1	No relevant information offered, repetition of sentences from the vignette
	2	Identification of only one of the options that choice entails
	3	Correct identification of the choice that must be made
Generation of alternatives (what possible choices does the protagonist have?)	1	No relevant information offered, no alternatives named on the vignette are mentioned
	2	Alternatives named in the story are mentioned
	3	Other possible alternatives, not named in the story, are mentioned
	Additional points	The total number of logical alternatives mentioned by the participant
Evaluation of alternatives (what are the advantages and disadvantages related to each choice?)	1	No relevant information is offered, not even one advantage and disadvantage are mentioned
	2	One advantage and one disadvantage related to each of the alternatives is mentioned
	3	More than one advantage and disadvantage related to each alternative is mentioned
	Additional points	The total number of advantages and disadvantages mentioned by the participant
Decision (what decision should the protagonist make and why?)	1	No relevant information is offered
	2	Decision is made but no logical justification is provided
	3	Decision is made and logical justification is provided
